# Extracting accurate PDF data from in situ environment of materials using X-ray diffractometer

**DOI:** 10.1007/s44211-025-00728-6

**Published:** 2025-02-13

**Authors:** Yuji Shiramata, Takayuki Konya, Kentaro Kobayashi, Yui Ishii, Satoshi Hiroi, Hiroki Yamada, Koji Ohara

**Affiliations:** 1 Graduate School of Natural Science and Technology, 1060, Nishikawatsu-cho, Matsue, Shimane 690-8504 Japan; 2 Product Division, Rigaku Corporation, 3-9-12, Matsubara-cho , Akishima-shi, Tokyo 196-8666 Japan; 3 Faculty of Materials for Energy, Shimane University, 1060, Nishikawatsu-cho, Matsue, Shimane 690-8504 Japan; 4 Co-Creation Institute for Advanced Materials, 1060, Nishikawatsu-cho, Matsue, Shimane 690-8504 Japan; 5 Diffraction and Scattering Division, Japan Synchrotron Radiation Research Institute, 1-1-1, Sayo-cho, Sayo-gun, Kouto, Hyogo 679-5198 Japan

**Keywords:** Pair distribution function, Synchrotron radiation, Laboratory-based equipment

## Abstract

Pair distribution function (PDF) analysis is a technique traditionally used at synchrotron radiation facilities, but it is now possible to obtain adequate measurements using laboratory-based equipment as well. In addition, cryo-furnaces that enable in situ measurements while controlling temperature have been well established for some time. PDF analysis requires data from a wide range of reciprocal space, along with measurements at short wavelengths. However, the development of time-resolved PDF measurements, which can be captured within a short time span, has also made significant progress. In this study, we demonstrate the reliability of PDF measurements using laboratory equipment and perform a local structure analysis of materials exhibiting an interesting structural phase transition in LaCu_6-*x*_Ag_*x*_ through temperature-dependent in situ PDF analysis. By comparing these results with those obtained from synchrotron sources, we provide further validation of the laboratory-based measurements. Our results confirm that in situ PDF measurements obtained with laboratory-based equipment are reliable and effective for studying structural disorder in various materials.

**Graphical abstract:**

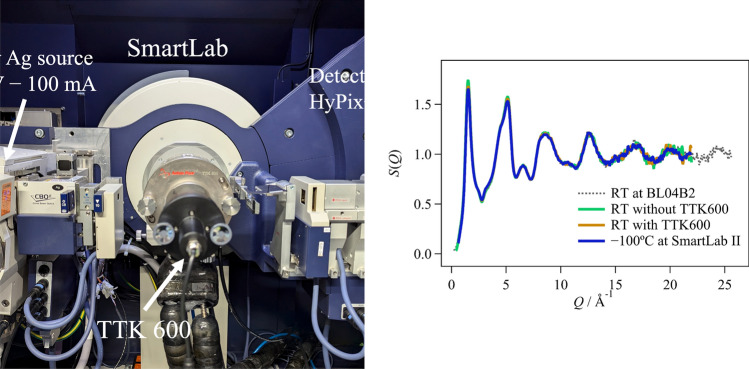

**Supplementary Information:**

The online version contains supplementary material available at 10.1007/s44211-025-00728-6.

## Introduction

The pair distribution function (PDF) is an important method that is used to obtain information about the interatomic distances of materials; it can be used to analyze the structures of materials, regardless of the crystallinity (order/disorder) of the material sample. The history of PDF analysis is quite long, as it dates back to 1927 when F. Zernike and others confirmed that the structure factor that was experimentally measured corresponded directly to the PDF [1].

Measurement data for PDF analysis requires a wide range of reciprocal space information as well as measurements at short wavelengths. For this reason, synchrotron radiation is often used to collect the measurement data, as it generates powerful coherent X-rays. The first synchrotron radiation PDF measurements were taken by Tsuyoshi Egami in the 1980s, overseas at the Cornell High Energy Synchrotron Source and the National Synchrotron Light Source at Brookhaven National Laboratory in the USA.

The development of time-resolved PDF measurement, which can be measured within a short time span, has also progressed. Since the beginning of 2000, many papers have been published on topics related to X-ray PDF [2, 3]. In Japan, K. Ohara et al. installed a time-resolved PDF measurement system at SPring-8 in 2018 and reported in detail the crystallization process of Li_7_P_3_S_11_, which is an important solid electrolyte material for all-solid-state lithium-ion batteries [4]. Their research produced important insights for improving the performance of lithium-ion batteries.

Recently, the technological capabilities of X-ray tubes and detectors have dramatically improved, and it has become possible to obtain data from laboratory-based equipment that is equivalent in quality to synchrotron radiation. This progress provides new opportunities to use PDF analysis on various materials using laboratory-based equipment in the same way as synchrotron radiation. For example, the Empyrean diffractometer from Malvern Panalytical uses a strip detector with a CdTe element that improves the absorption efficiency of high-energy diffraction lines [5]. STOE company’s STADIP diffractometer is equipped with AgKα_1_ to improve its spatial resolution [6], Bruker’s D8 Advance diffractometer is equipped with Dectris’ Eiger 2R 500 K [7], and the RAPID II diffractometer from Rigaku is a two-dimensional (2D) measurement system that uses imaging plates to enable wide-area, short-time, 2D measurements [8]. Until recently, powder structural analysis by laboratory equipment was mainly carried out by Rietveld refinement, which can only be used on crystalline materials, but PDF analysis has been attracting attention as an effective method for the structural analysis of complex material systems because it can be used on materials regardless of their crystallinity, and the usefulness of laboratory PDF equipment has already been reported for amorphous silicon [9]. Furthermore, in situ measurement technology has also been established for laboratory-based equipment, which involves collecting measurement data under conditions of changing temperature. This makes it possible to evaluate materials by capturing low-temperature measurements [10] with liquid helium or liquid nitrogen and capturing high-temperature measurements using heating elements such as nichrome wire or platinum wire [11, 12], as well as to understand the behavior of materials under various conditions. However, there are few examples of in situ measurements and PDF analysis being applied in laboratory systems. The main reason for this scarcity is that the furnace body emits parasitic scattered radiation. For example, Anton Paar’s furnace body is provided with a knife edge tool, which suppresses scattering, but because it is not possible to measure at high angles of diffraction, it is not possible to obtain the PDF measurement data.

For this study, we first compared the synchrotron radiation PDF patterns with those from laboratory-based systems and verified the validity of the patterns using silica glass. This showed that the analysis could be performed without problems. We also verified the validity of in situ PDF measurements that suppress parasitic scattering from the furnace body. Our results confirmed that in situ PDF measurements that we captured using laboratory-based equipment were reliable. We demonstrate that measurements can be taken without problems, even when the temperature is changing, and these measurements form the basis for future materials research. As a suitable compound to prove the usefulness of our system, we selected the LaCu_6-*x*_Ag_*x*_ intermetallic compound, which exhibits an interesting structural phase transition [13]. The parent compound LaCu_6_ is known to crystallize into an orthorhombic structure at high temperatures. It undergoes the structural phase transition at *T*_s_ = 470 K, below which the crystal structure transforms to a monoclinic structure. The transition temperature decreases with the Ag composition *x*, and disappears at *x* = 0.22, indicating that the structure remains orthorhombic at all temperatures at compositions larger than *x* = 0.22 [13].

### Sample preparation

A 0.5 mmφ rod of SiO_2_ quartz glass having a purity of 99.9%, made by Nakahara Opto-Electric Lab, was measured using synchrotron radiation and laboratory equipment. The LaCu_6-*x*_Ag_*x*_ samples for the in situ PDF measurements were prepared as follows. Pure elements of La, Cu, and Ag at a ratio of 1:6-*x*:*x* (*x* = 0.125, 0.4) were melted in a monoarc furnace to obtain a uniform mixture. The obtained ingot was put in an evacuated silica tube and annealed at 750 °C for 15 h. After annealing, the ingot was crushed into powder and sieved to obtain a fine powder with minimal distortion.

### X-ray PDF measurement

We used the SmartLab, which is a fully automated multipurpose X-ray diffractometer, as the laboratory equipment. The optical system used a capillary for transmission. The X-ray source was AgKα (*λ* = 0.561 Å, and *E* = 21.986 keV); the measurement range was 2*θ* = 3.0–155.0° (*Q* = 0.587 to 21.877 Å^−1^); the scattering interval was *Δ*2*θ* = 0.2° (*Q* = 0.04 Å^−1^); the exposure time was 1 min per the scattering angle; and a HyPix-3000 HE 2D detector with a thick silicon element was used as the detector.

Measurements at the synchrotron radiation facility were carried out using 113.2 keV X-rays at BL04B2 of SPring-8, with a measurement range of 2*θ* = 0.3–24.7° (*Q* = 0.3 to 24.3 Å^−1^); the scattering interval was *Δ*2*θ* = 0.05° (*Q* = 0.05 Å^−1^); and the exposure time was 15 s per the scattering angle. A triple Ge semiconductor detector was used as the detector [14].

The intensity of the X-ray scattering comprises the three components shown in the following Eq. (1). These are coherent scattering, whose source is the structure of the material; incoherent scattering, whose source is the constituent atoms of the material and is also known as Compton scattering, and fluorescent X-rays, which are generated by the combination of the constituent elements of the material and the energy of the incident X-rays:

1 $$I_{obs} = I_{coh} + I_{incoh} + I_{XRF}$$

Since the coherent scattering intensity is required here, the incoherent scattering and fluorescence X-rays must be subtracted (removed) with the use of a detector discriminator or crystal monochromator.

### In situ measurement of cooling and heating

**Fig. 1:**
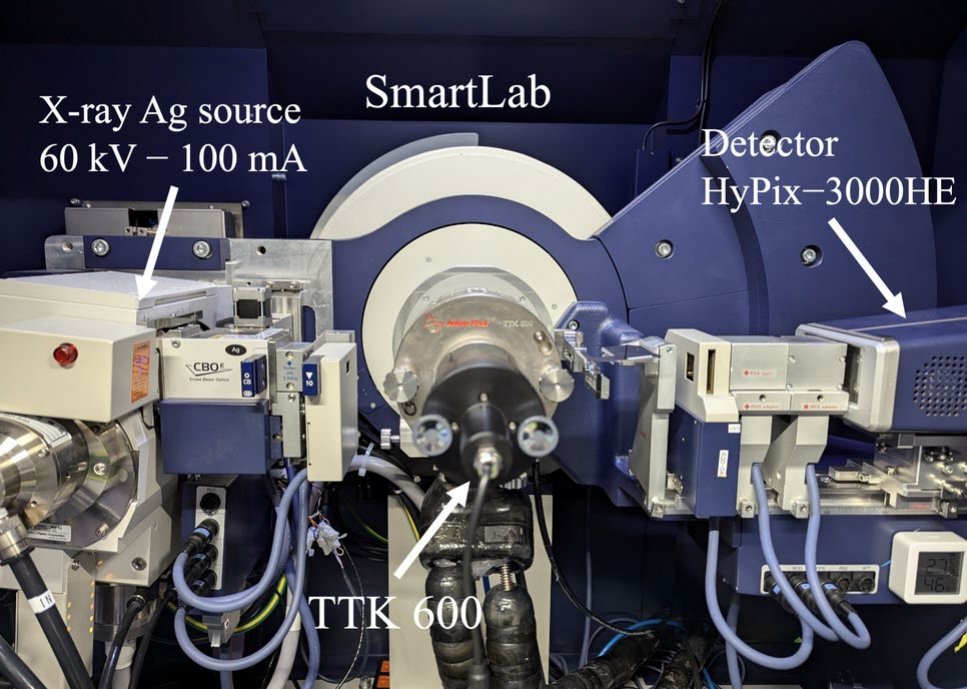
SmartLab with TTK600 cryo-furnace

To obtain the cooling and heating measurements, we used the TTK600 cryo-furnace manufactured by Anton Paar. The TTK600 achieves high-precision temperature control using liquid nitrogen and resistance heating and can be set in the temperature range of − 190 to 600 °C. Measurements were carried out using a capillary sample holder with a rotation system, and measurements of the total scattering were taken at RT and at − 100 °C. By rotating the capillary, we were able to average the intensity by exposing the sample to X-rays from various crystal planes, even though the sample contained a coarse grain, as shown in Fig. S1. The system that includes both SmartLab and TTK600 is shown in Fig. 1.

### PDF analysis

We used the TXS plug-in for Rigaku’s SmartLab Studio II software to measure the structure factor *S*(*Q*) and the reduced PDF, *G*(*r*), from the X-ray scattering obtained. The *S*(*Q*) is expressed by the following Eq. (2), where the incoherent scattering component$I_{incoh}$ is subtracted from the raw data to obtain the coherent scattering component $I_{coh}$, which is then normalized by the atomic form factor:

2 $$S\left( Q \right) = \frac{{\frac{{I_{coh} \left( Q \right)}}{N} - \left[ {\left( {\mathop \sum \nolimits_{i} c_{i} f_{i}^{2} } \right) - \left( {\mathop \sum \nolimits_{i} c_{i} f_{i} } \right)^{2} } \right]}}{{\left( {\mathop \sum \nolimits_{i} c_{i} f_{i} } \right)^{2} }},$$

3 $$Q = \frac{4\pi \sin \theta }{\lambda },$$

where $I_{{{\text{coh}}}}$ is the coherent scattering component, *N* is the number of atoms, *c*_*i*_ is the atomic fraction of chemical species *i*, *f*_*i*_ is the atomic form factor for each chemical species, and *Q* is the scattering vector magnitude. The reduced PDF, *G*(*r*), is obtained by applying a Fourier transform to *S*(*Q*). The formula is as follows:

4 $$G\left( r \right) = \frac{2}{\pi }\mathop \int \limits_{{0\left( {Q_{min} } \right)}}^{{\infty \left( {Q_{max} } \right)}} Q\left[ {S\left( Q \right) - 1} \right]\sin Qr\;dQ$$

### Rietveld refinement

Rietveld refinement was carried out using the powder plug-in included with Rigaku’s SmartLab Studio II software. This refinement is a method that is widely used to analyze the crystal structure of powders. We used this method to analyze the average structure of LaCu_6-*x*_Ag_*x*_. The accuracy of the analysis was evaluated using the numerical values of the *R*-weighted pattern, *R*_wp_, as shown in the following equation:

5 $${R}_{\text{wp}}={\left[\frac{\sum_{i}{w}_{i}{\left\{{y}_{i}-{f}_{i}(x)\right\}}^{2}}{\sum_{i}{w}_{i}{y}_{i}^{2}}\right]}^{1/2}$$

where *w*_*i*_ is the statistical weight of the *i*-th diffraction intensity, *y*_*i*_ is the experimental diffraction intensity, and *f*_*i*_(*x*) is the theoretical diffraction intensity by Rietveld refinement.

### PDFgui

PDFgui was developed by S. Billinge et al. to enable the extraction of local structural information. It refines the theoretical PDF pattern calculated from the structural model using the structural parameters for the measured PDF pattern [15]. This method is well known as the “small box approach”. We used it to analyze the local structure of LaCu_6-*x*_Ag_*x*_. The accuracy of the analysis was evaluated using the residual function, *R*_w_. This is almost the same as the *R*_wp_ of the Rietveld analysis, but it was evaluated using PDF pattern, *G*(*r*), as shown in the following equation:

6 $${R}_{\text{w}}={\left[\frac{\sum_{i}w({r}_{i}){\left\{{G}_{Exp.}({r}_{i})-{G}_{Model}({r}_{i})\right\}}^{2}}{\sum_{i}{w(r}_{i}){G}_{Exp.}^{2}({r}_{i})}\right]}^{1/2}$$

where the weight *w*(*r*_*i*_) is set to unity which is justified because in *G*(*r*) the statistical uncertainty on each point is approximately equal [16, 17].

## Results and discussion

### Comparison between *S*(*Q*)s measured at SPring-8 and at SmartLab

**Fig. 2:**
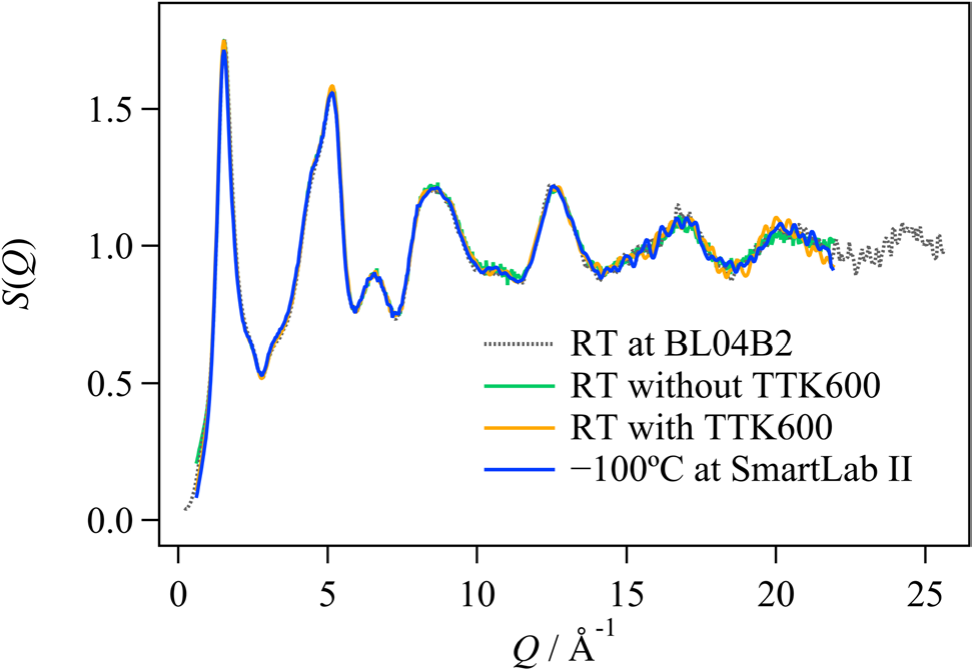
Comparison of the *S*(*Q*) values in the SiO_2_ glass: black dotted line, room temperature (RT) at BL04B2 in SPring-8; green solid line, RT at SmartLab without the TTK600 furnace; orange solid line, RT at SmartLab with the TTK600 furnace; blue solid line, − 100 °C at SmartLab with the TTK600

The total X-ray scattering for SiO_2_ glass and for LaCu_6-*x*_Ag_*x*_ was measured using BL04B2 at SPring-8 and at SmartLab at Shimane University. The *S*(*Q*) data for SiO_2_ glass are shown in Fig. 2, and the corresponding *G*(*r*) are shown in Fig. S2. Although the *Q* range is quite limited at SmartLab, there was consistency up to a *Q* range of 22 Å^−1^. Furthermore, it was found that the same values were obtained for *S*(*Q*) when the TTK600 cryo-furnace was attached as when it was not attached, and when the temperature was set to room temperature (RT) or to − 100 °C using the TTK600 cryo-furnace. From these results, it can be said that the measurement data for the total X-ray scattering obtained by attaching the TTK600 cryo-furnace to the SmartLab laboratory system is reliable. Using a scattering protector instead of the knife edge provided with the TTK600, we successfully measured at high angles, as shown in Fig. S3.

### In situ PDF measurement of LaCu_6-*x*_Ag_*x*_ using a combination of TTK600 and SmartLab

**Fig. 3:**
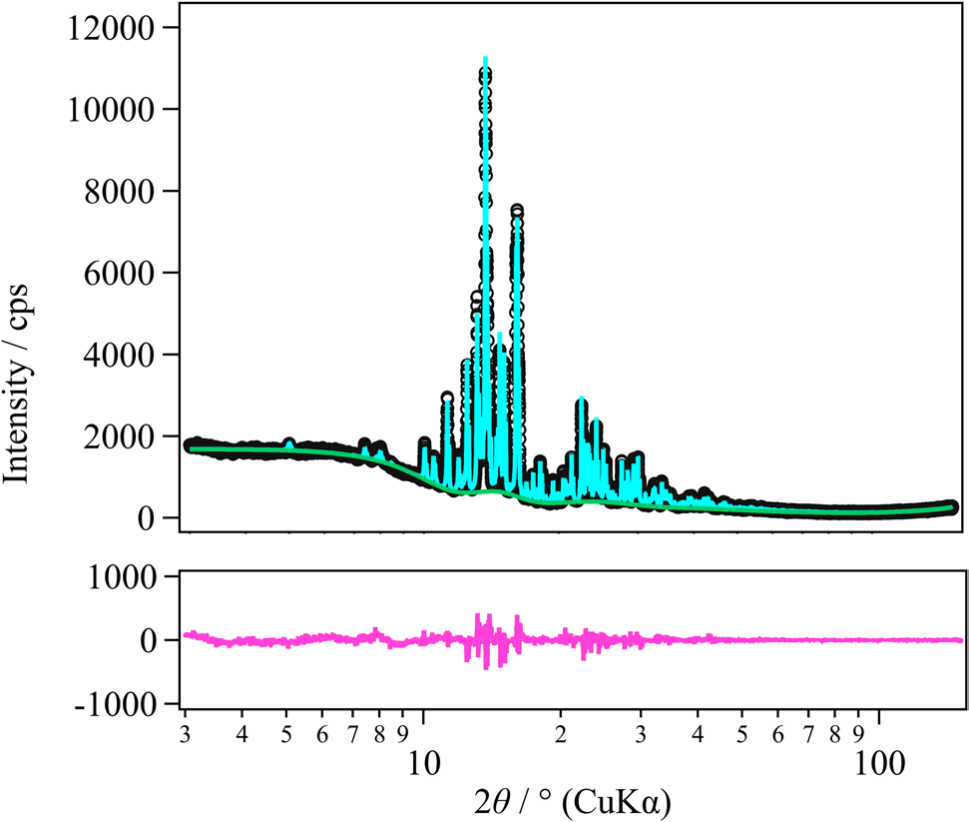
X-ray diffraction pattern and Rietveld refinement in the orthorhombic phase of the composition LaCu_6-*x*_Ag_*x*_*, x* = 0.125 at RT: black open circles, experimental X-ray diffraction pattern for* x* = 0.125; light blue solid line, Rietveld refinement for* x* = 0.125; green solid line, the background; pink solid line, the difference between the experimental and refinement results

To give an example of the results obtained, Fig. 3 shows the results for refining the orthorhombic phase for* x* = 0.125 measured at RT, and Table 1 shows the results for fitting the orthorhombic phase and the monoclinic phase using the Rietveld method for each composition and temperature. According to the previous reports [13], the *x* = 0.125 sample exhibits a clear structural phase transition at *T*_s_ = 200 K from the orthorhombic structure to the monoclinic structure. On cooling, the Bragg reflection in the orthorhombic phase split into two monoclinic reflections, as seen in the 122, 220, and 221 reflections in the literature. These peak splittings have also been confirmed in our diffractometer. On the other hand, such the peak splitting has not been observed in the *x* = 0.4 sample at − 100 °C, indicating that the sample remains orthorhombic in the average structure.

**Table 1:** Rietveld refinement for the compositions *x* = 0.125 and *x* = 0.4 at RT and at − 100 °C in the orthorhombic and monoclinic phases

Composition *x*	Temperature	*R*_wp_ (%) in orthorhombic phase	*R*_wp_ (%) in monoclinic phase
0.125	**RT**	5.1	5.2
0.4		4.7	5.0
0.125	**− 100 °C**	8.3	5.1
0.4		4.6	4.6

Table 1 summarizes the results of the Rietveld refinements for *x* = 0.125 and 0.4 samples, respectively. As shown in the table, the refinements for each temperature data using each structure model yield good *R*_wp_ values both for *x* = 0.125 and 0.4 samples, consistent with the previous report [13]. Because of the structural phase transition of *x* = 0.125, the refinement using the monoclinic structure model provides a better agreement for the *x* = 0.125 sample than that using the orthorhombic structure model.

**Fig. 4:**
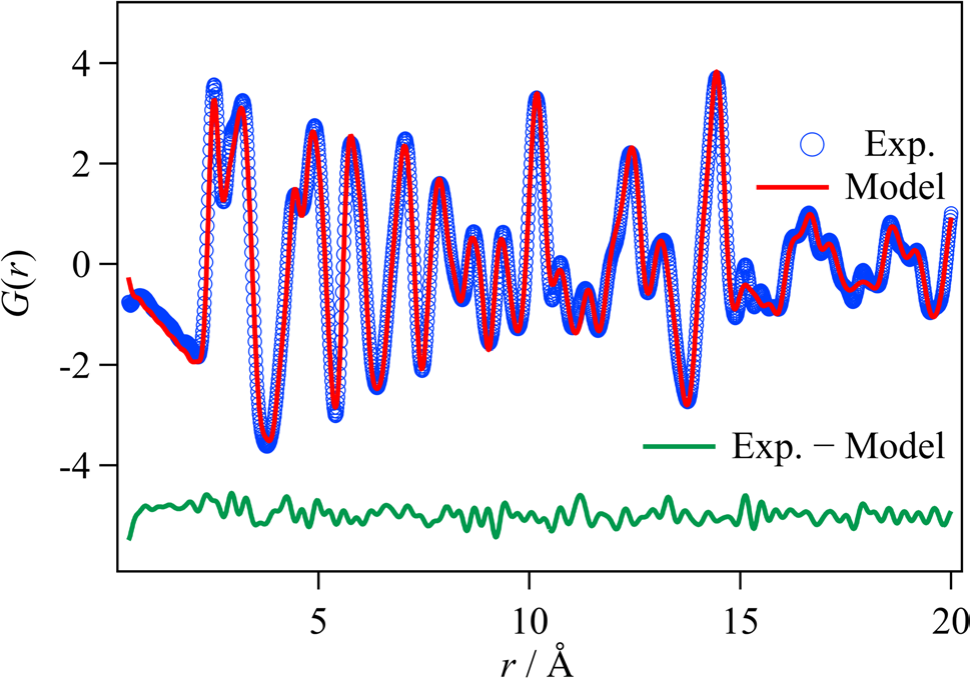
Comparison of the *G*(*r*) values in the LaCu_6-*x*_Ag_*x*_ using PDFgui with the composition *x* = 0.125 in the orthorhombic phase at RT

Local structure refinement was performed using PDFgui. The refinement range was set to 0.5–20.0 Å for the short-range side, which reflects the local structure, and to 20.0–40.0 Å for the long-range side, which reflects the average structure. Figure 4 shows the result of the refinement using PDFgui for the orthorhombic phase and room temperature, and Table 2 shows the *R*_w_ values for each composition and temperature.

**Table 2:** PDFgui refinement for the compositions *x* = 0.125 and *x* = 0.4 at RT and at − 100 °C in the orthorhombic and monoclinic phases

Composition *x*	Temperature	*R*_w_ (%) in orthorhombic phase	*R*_w_ (%) in monoclinic phase
**0.125**	**RT**	9.9 (0.5–20.0 Å)	10.0 (0.5–20.0 Å)
		13.7 (20.0–40.0 Å)	14.0 (20.0–40.0 Å)
**0.4**	**RT**	9.4 (0.5–20.0 Å)	8.6 (0.5–20.0 Å)
		12.6 (20.0–40.0 Å)	12.5 (20.0–40.0 Å)
**0.125**	**− 100 °C**	10.3 (0.5–20.0 Å)	8.4 (0.5–20.0 Å)
		14.2 (20.0–40.0 Å)	14.0 (20.0–40.0 Å)
**0.4**	**− 100 °C**	8.9 (0.5–20.0 Å)	7.5 (0.5–20.0 Å)
		11.9 (20.0–40.0 Å)	11.6 (20.0–40.0 Å)

From the *R*_w_ values for *x* = 0.125 at RT, it was found that the local and the average structures were orthorhombic phase, and they were the monoclinic phase at − 100 °C, reflecting that the reported crystal structures are orthorhombic and monoclinic at RT and − 100 °C, respectively.

For *x* = 0.4, it was suggested that the local structure assumes the monoclinic phase, although the average structure assumes the orthorhombic phase at both temperatures. In the refinement of a fitting range of *r* = 20.0 − 40.0 Å, the difference between the *R*_w_ values of orthorhombic and monoclinic structures is small. These results imply that local structure distortion exists in the *x* = 0.4 owing to suppression of the structural phase transition. Finally, the crystal systems for each composition and temperature inferred from these results are summarized in Table 3.

**Table 3:** PDFgui refinement for LaCu_6-*x*_Ag_*x*_

Composition *x*	Temperature	Average	Local
0.125	RT	Orthorhombic	Orthorhombic
0.4	RT	Orthorhombic	Monoclinic
0.125	**− **100 °C	Monoclinic	Monoclinic
0.4	**− **100 °C	Orthorhombic	Monoclinic

## Conclusion

In this study, we demonstrated the validity of the laboratory PDF data by comparing them with the synchrotron radiation PDF data. Furthermore, we found that reliable PDF data and local structural information could be measured at low temperatures (− 100 °C) and RT (up to high temperatures 600 °C from the furnace’s performance) for in situ PDF measurements that suppressed parasitic scattering with the SmartLab system. In addition, as powder X-ray diffraction data can also be obtained simultaneously with SmartLab, it can be said that the realization of this system would provide precise analyses of both local and average structures.

## Supplementary Information

Below is the link to the electronic supplementary material.

Supplementary file1 (DOCX 878 KB)

## Data Availability

The datasets generated and/or analyzed during the current study are available from the corresponding author on reasonable request.

## References

[1] F. Zernike, J.A. Prins, Z. Phys. **41**, 184–194 (1927)

[2] S. Billinge, Phil. Trans. R. Soc. A **377**, 1–17 (2018)

[3] A.K. Soper, E.R. Barney, J. Appl. Cryst. **44**, 714–726 (2011)

[4] K. Ohara, S. Tominaka, H. Yamada, M. Takahashi, H. Yamaguchi, F. Utsuno, T. Umeki, A. Yao, K. Nakada, M. Takemoto, S. Hiroi, N. Tsujia, T. Wakihara, J. Synchrotron Rad. **25**, 1627–1633 (2018) 10.1107/S1600577518011232 PMC6225740 30407171

[5] J. Bolze, M. Gateshki, Rev. Sci. Instrum. **90**, 123103 (2018) 10.1063/1.5130061 31893848

[6] L.J. Sabrina, N. Thomae, T. Prinz, M. Hartmann, S. Teck, M. Correll, M. Zobel, Rev. Sci. Instrum. **90**, 043905 (2019) 31043011 10.1063/1.5093714

[7] D. Tsymbarenko, D. Grebenyuk, M. Burlakova, M. Zobel, J. Appl. Cryst. **55**, 890–900 (2022)

[8] D.J.M. Irving, D.A. Keen, M.E. Light, Rev. Sci. Instrum. **92**, 043107 (2021) 34243411 10.1063/5.0040694

[9] R.K. Biswas, P. Khan, S. Mukherjee, A.K. Mukhopadhyay, J. Ghosh, K. Muraleedharan, J. Non-Cryst Solid. **488**, 1–9 (2018)

[10] A. Zandona, G. Helschand, J. Deubener, J. Am. Ceram. Soc. **103**, 6630–6638 (2020)

[11] L. Zhou, X. Ge, C. Ren, G. Chen, J. Alloys Comp. **778**, 72–76 (2019)

[12] A. Moatti, R. Sachan, V. Cooper, J. Narayan, Sci. Rep. **9**, 1–10 (2019) 30816206 10.1038/s41598-019-39529-z PMC6395818

[13] L. Poudel, C. de la Cruz, M.R. Koehler, M.A. McGuire, V. Keppens, D. Mandrus, A.D. Christianson, Phys. B Condens. Matter. **536**, 479–482 (2018)

[14] K. Ohara, Y. Onodera, M. Murakami, S. Kohara, J. Phys. Condens. Matter **33**, 383001 (2021) 10.1088/1361-648X/ac0193 34286699

[15] P. Juhas, C. Farrow, X. Yang, K. Knox, S. Billinge, Acta Cryst. **A71**, 562–568 (2015) 10.1107/S2053273315014473 26522405

[16] B. Toby, T. Egami, Acta Crystallogr. A **48**, 336 (1992)

[17] B. Toby, S. Billinge, Acta Crystallogr. A **60**, 315 (2004) 15218209 10.1107/S0108767304011754

