# Supplementary material for: Extracting accurate PDF data from in situ environment of materials using X-ray diffractometer
Source: Anal Sci. 2025 Feb 13;41(6):777–82. doi: 10.1007/s44211-025-00728-6 (PMC12137423; doi:10.1007/s44211-025-00728-6)
Supplement: Supplementary file 1 — Supplementary file1 (DOCX 878 KB) [file 44211_2025_728_MOESM1_ESM.docx]

**Supporting data**

**
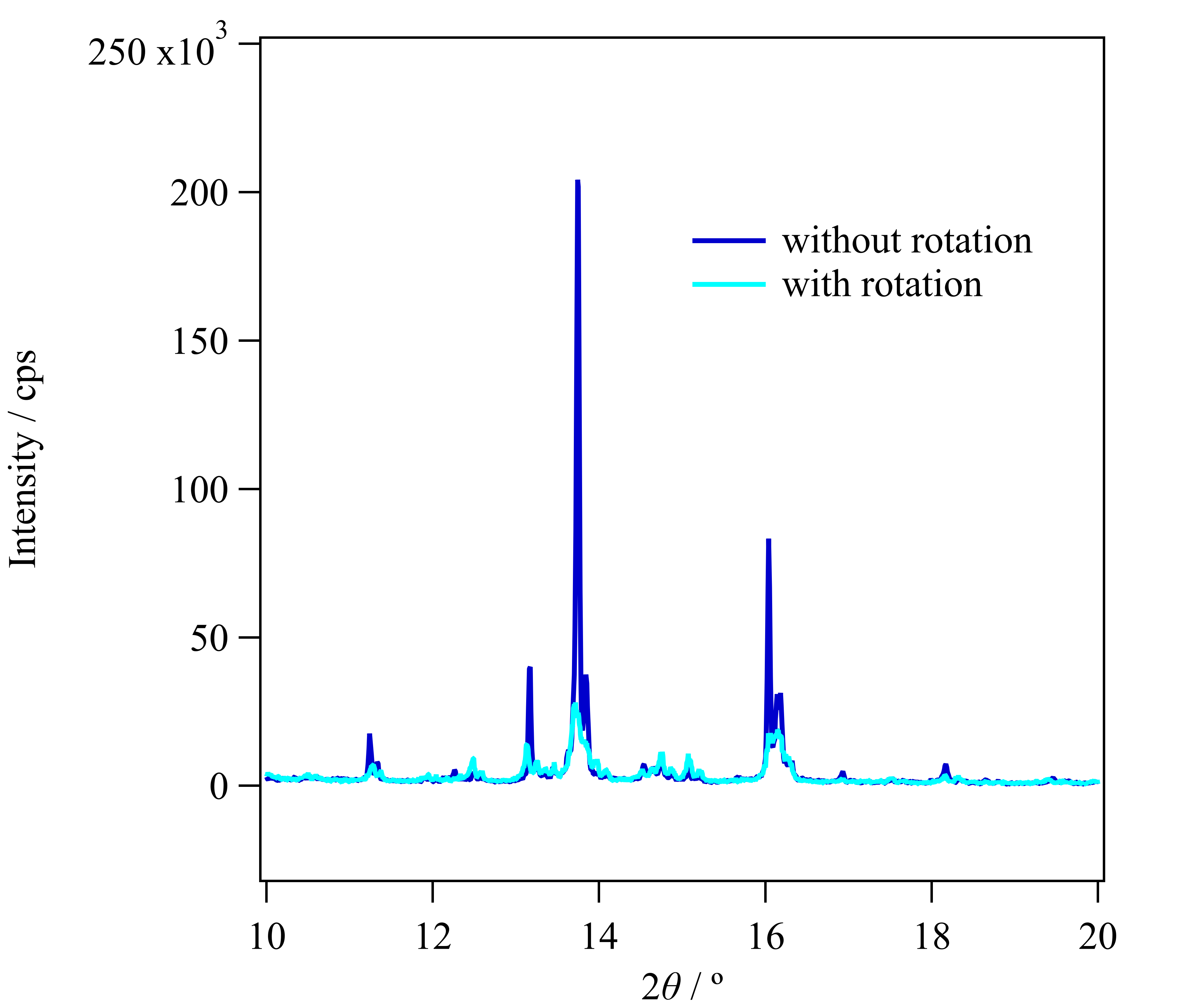
**

**Fig. S1** Scattering intensity with and without the rotation of the TTK600 cryo-furnace

**
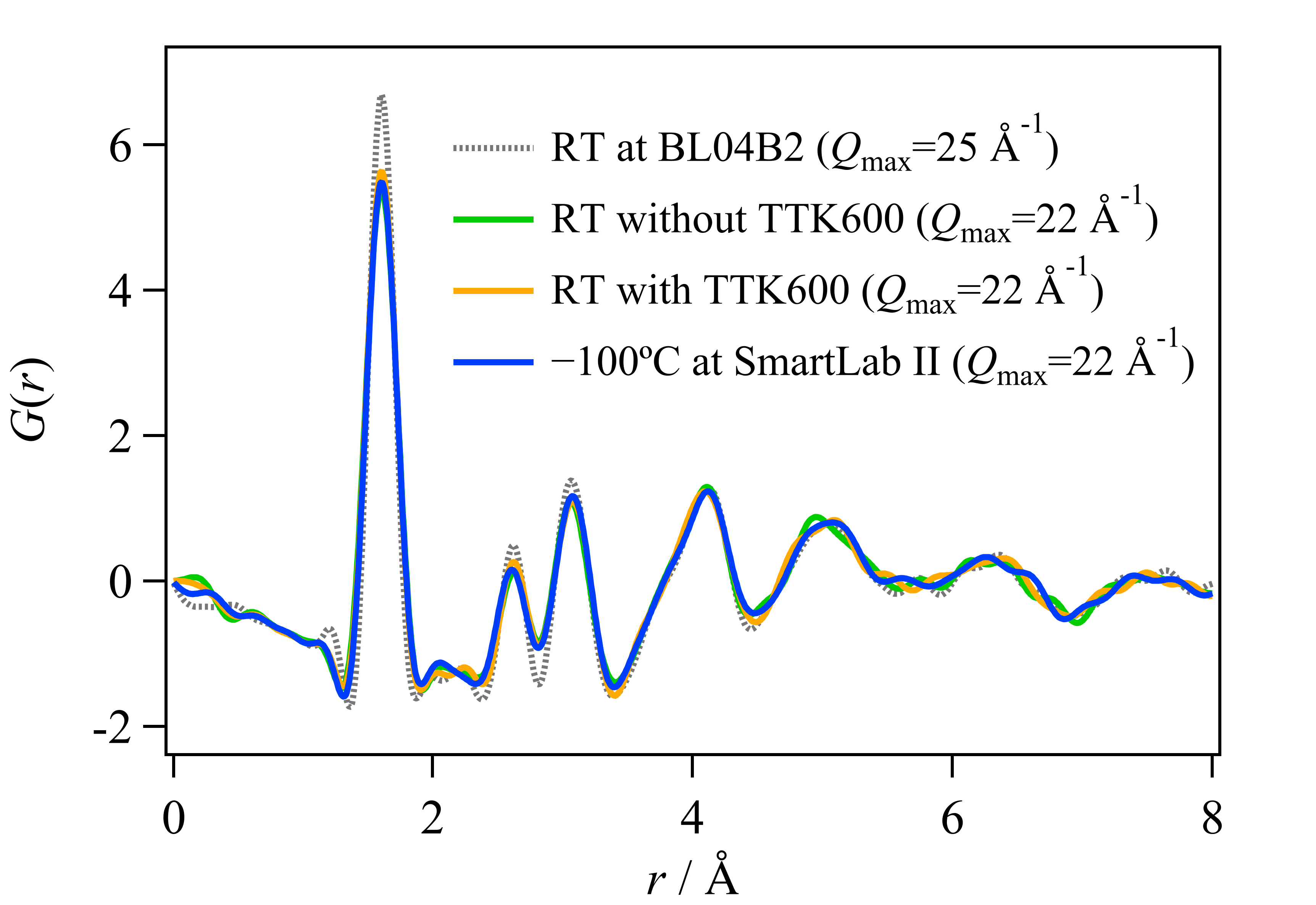
**

**Fig. S2** *G*(*r*) pattern of SiO_2_ measured at BL04B2 of SPring-8 and SmartLab: black dotted line, room temperature (RT) at BL04B2 in SPring-8; green solid line, RT at SmartLab without the TTK600 furnace; orange solid line, RT at SmartLab with the TTK600 furnace; blue solid line, -100°C at SmartLab with the TTK600

**
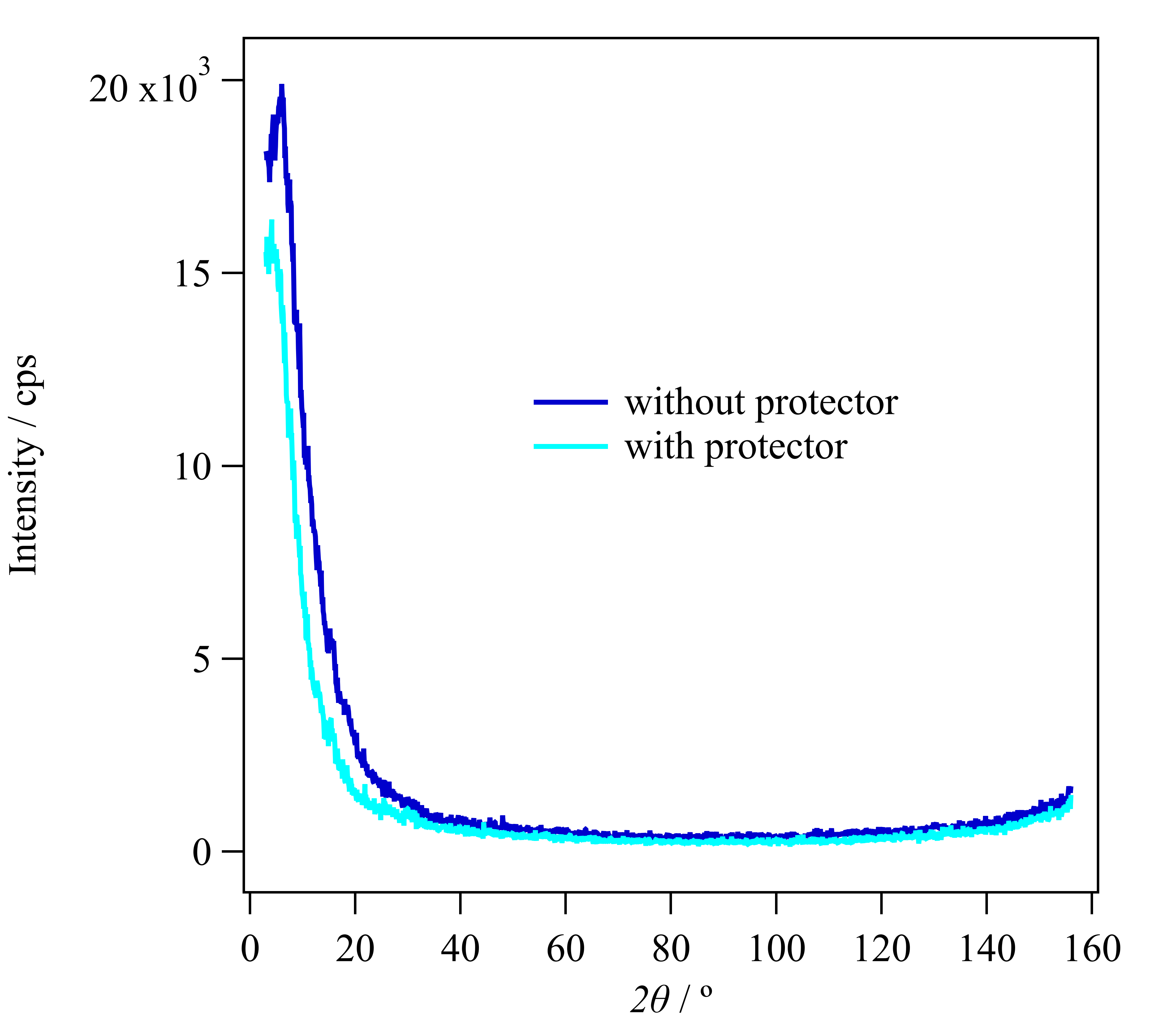
**

**Fig. S3** Air scattering measured with and without the scattering protector at SmartLab
